# Validating an Ultrasonographical Assessment Tool for Predicting Difficult Laparoscopic Cholecystectomy in a Tertiary Care Hospital

**DOI:** 10.7759/cureus.52048

**Published:** 2024-01-10

**Authors:** Hira Bakhtiar Khan, Aiman Ali, Muhammad Jawad Zahid, Sana Hamayun, Abdul Haseeb, Ahmad Shiraz, Maryam Karim, Fawad Ali, Aimon Akhtar, Qaidar Alizai

**Affiliations:** 1 General Surgery, Hayatabad Medical Complex Peshawar, Peshawar, PAK; 2 General Surgery, Rehman Medical Institute, Peshawar, PAK

**Keywords:** ultrasonological assessment tool, laparoscopy, cholelithiasis, difficult laparoscopic cholecystectomy, laparoscopic cholecystectomy

## Abstract

Background: Laparoscopic cholecystectomy (LC) is the preferred treatment option in non-complicated symptomatic cholelithiasis. In some cases, the surgery might be complicated by different factors resulting in difficult LC. Ultrasound remains the first-line modality for diagnosing symptomatic cholelithiasis; however, its role in predicting difficult LC remains unclear. The aim of this study was to validate an ultrasonographic scoring system in predicting difficult LC.

Methods: We prospectively enrolled patients undergoing LC in a tertiary care unit over six months. All adult (≥18 years) patients undergoing LC for symptomatic cholelithiasis were included. Patients were excluded if they refused to consent, and those who underwent cholecystectomy for indications other than cholelithiasis. Patients were stratified into two groups based on intra-operative difficulty (easy LC and difficult LC) and were compared. Our primary outcome was radiologic difficulty among these groups. Univariate analysis and kappa statistics were performed.

Results: We identified 68 patients with an overall mean (SD) age of 42.2 (12.3) years, a mean (SD) weight of 74.1 (10.9) kg, and 73.5% were female. Of the study cohort, 52 patients had easy LC and 16 patients experienced difficult LC. Amongst the total, 14.7% suffered from diabetes mellitus, 29.4% had hypertension, 7.4% had a known ischemic heart disease, and 63% had a body mass index (BMI) ≥30 kg/m^2^ with no statistically significant difference between the two groups. On the Chi-square test, there was no statistical difference between the two groups in terms of ultrasonographic difficulty (p>0.05). However, we found a Kappa value of -0.127 (p=0.275) corresponding to a strong disagreement between the intraoperative and ultrasonographic difficulty.

Conclusion: Despite its role in diagnosing cholelithiasis, an ultrasonographic assessment did not have a role in predicting difficult LC according to the present study. Further studies are required to develop a scoring system for predicting difficult LC based on clinical, laboratory, and ultrasonographic assessment.

## Introduction

Gallstone disease, also known as cholelithiasis, represents a significant global health burden [1]. Most of the patients have asymptomatic cholelithiasis and do not require any treatment [2]. However, a minority of patients present with severe or recurrent symptoms or signs of cholecystitis requiring conservative management with medications, endoscopic cholangiopancreatography (ERCP), percutaneous drainage, or operative treatment [3]. Laparoscopic cholecystectomy (LC) is the most commonly used and preferred operative management for patients with recurrent symptomatic cholelithiasis [4,5]. This approach offers advantages including reduced postoperative pain, quicker recovery, hastened bowel function return, and shorter hospital stay [6-8].

LC is uncomplicated in most cases, but some patients might have difficult anatomy of the gallbladder bed, anomalous ducts, and vessels, or dense adhesions that make the identification and resection complicated [9]. This can result in severe morbidity and mortality both during and after the surgery. Difficult LC has been defined based on operative duration exceeding 60 minutes, injury to cystic duct or artery, presence of biliary leakage, and need for conversion to open cholecystectomy for any reason [10]. Both surgeon and patient factors have been reported to contribute to difficult LC [11-13]. These include male gender, old age (>60 years), history of abdominal surgery, gallbladder wall thickness ≥4-5 mm, impacted stones, fibrotic gallbladder, adhesions at Calot's triangle, and less-experienced surgeons [5,14]. Despite these known risk factors, pre-operative clinical identification of patients at risk of difficult cholecystectomy remains challenging, particularly in developing countries. Although ultrasonography has been shown to predict difficult LC in some studies, there is a paucity of studies on this subject in developing countries. The aim of our study was to analyze the validity of ultrasonography-based scoring in predicting difficult LC in a developing country.

## Materials and methods

This was a cross-sectional study conducted on patients undergoing LC in the Department of General Surgery of Hayatabad Medical Complex, Peshawar, over six months from June 1, 2022, to December 31, 2022. Using an anticipated frequency of 78.8% [10], the sample size was calculated to be a minimum of 56. These calculations were made using the OpenEpi calculator with a 90% confidence interval and a 10% margin of error [15]. Employing convenience sampling, 68 patients were included in the study. All adult patients (age ≥ 18 years) who underwent LC ultrasound-confirmed symptomatic gallstones during the study period were included. We excluded patients who refused to consent to participate in the study and also those who underwent cholecystectomy for indications other than cholelithiasis.

The study cohort was stratified into two groups based on intra-operative difficulty: (i) Easy LC and (ii) Difficult LC. Difficult LC was defined based on the presence of any of the following criteria: Conversion to open cholecystectomy, Operative duration exceeding 60 minutes, Vascular and/or biliary injuries, significant bleeding and/or use of synthetic hemostats. Our primary outcome included rates of difficult laparoscopic cholecystectomies based on ultrasonographical prediction.

Institutional Review Board (IRB), Hayatabad Medical Complex, Peshawar, Pakistan, approved the study (approval number: 1130) After IRB approval and written informed consent, patients or their family members were approached for data collection. The tool used for data collection was a six-point ultrasonographic scoring chart, a modified form of the same tool employed by Siddique et al. [10], along with the modified Nassar scale [16], a recently introduced intra-operative difficulty grading system for LC. Our data included patient demographic characteristics (age and gender), comorbid conditions (diabetes mellitus, hypertension, obesity (defined as BMI ≥30 kg/m^2^), presenting signs and symptoms, concurrent pancreatitis, history of endoscopic cholangiopancreatography (ERCP), ultrasonographic characteristics, interventions performed, mortality, in-hospital complications, and hospital length of stay. We also collected data on laboratory findings including total leukocyte count, serum bilirubin level, serum alanine transaminase (ALT) level, serum alkaline phosphatase (ALP), and serum C-reactive protein (CRP) level. Univariate analyses were performed, and the two groups were compared in terms of baseline demographic and disease characteristics.

Continuous normally distributed variables were reported as means and standard deviations (SD). Chi-square and Fisher Exact tests were performed for categorical variables. The two groups were compared in terms of outcomes including Ultrasonographic difficulty using the Chi-square test. Moreover, the kappa coefficient was used to report the concordance between the intra-operative and ultrasonographic difficulty of our cohort. A p-value of <0.05 was considered statistically significant. All analyses were conducted using IBM SPSS Statistics for Windows, Version 25.0 (Released 2017; IBM Corp., Armonk, New York, United States).

## Results

Sixty-eight patients were identified based on our inclusion and exclusion criteria. The mean (SD) age was 42.2 (12.3) years, 73.5% were female, and the mean (SD) weight was 74.1 (10.9) kg. Overall, 52 patients had easy LC and 16 patients had difficult LC. Amongst the total, 14.7% suffered from diabetes mellitus, 29.4% had hypertension, 7.4% had a known ischemic heart disease, and 63% had a BMI ≥30 kg/m^2^. Furthermore, 89.7% experienced right hypochondriac pain and Murphy’s sign was positive on presentation in 39.7%. Concurrent pancreatitis was present in 23.5% of patients and three (4.4%) reported a history of ERCP. Laboratory findings indicated an ongoing inflammatory process with an average total leukocyte count of 9.45, a total bilirubin level of 0.7, an ALT level of 60, an ALP level of 134.4, and a CRP level of 2.57. Over half of the patients (57.4%) had a post-operative hospital stay of one day. There was no difference in the baseline demographic and disease characteristics between the two groups (p>0.05) except serum bilirubin and serum CRP levels, which were significantly high in the difficult LC group. Table 1 summarizes the baseline and disease characteristics.

**Table 1:** Comparison of Baseline Characteristics of Patients With Intra-operative Easy LC Versus Those With Difficult LC ALP= Alkaline Phosphatase; ALT= Alanine Transaminase; CRP= C-reactive Protein; ERCP= Endoscopic Cholangiopancreatography; LC= Laparoscopic Cholecystectomy; n (%) = Count (Percentage); LOS= Length of Stay; RHC= Right Hypochondrium; SD= Standard Deviation, TLC= Total Leukocyte Count *= Bold p-values indicate statistical significance

Variable	Total	Easy LC, n = 52	Difficult LC, n =16	p-value*
Age, mean (SD)	42.2 (12.34)	41.2 (12.9)	45.5 (9.6)	0.203
Female, n (%)	50 (73.5)	41 (78.8)	9 (56.3)	0.073
Weight, mean (SD)	74.1 (10.94)	73.8 (11.1)	75.0 (10.6)	0.265
Comorbidities, n (%)				
Diabetes Mellitus	10 (14.7)	10 (19.2)	0	0.058
Hypertension	20 (29.4)	17 (32.7)	3 (18.8)	0.284
Ischemic Heart Disease	5 (7.4)	5 (9.5)	0	0.330
Presenting Signs and Symptoms, n (%)				
RHC Pain	61 (89.7)	47 (90.4)	14 (87.5)	0.740
Vomiting	27 (39.7)	21 (40.4)	6 (37.5)	0.837
Fever	17 (25.0)	15 (28.8)	2 (12.5)	0.187
Murphy Sign	27 (39.7)	19 (36.5)	8 (50.0)	0.336
Pancreatitis	16 (23.5)	14 (26.9)	2 (12.5)	0.234
ERCP History	3 (4.4)	1 (1.9)	2 (12.3)	0.072
Laboratory Investigations, mean (SD)				
TLC	9.45 (2.6)	9.4 (2.8)	9.3 (2.1)	0.583
Total Bilirubin	0.7 (1.35)	0.5 (0.2)	1.5 (2.6)	<0.001
Serum ALT	60 (77.7)	61.0 (81.8)	56.3 (64.4)	0.538
Serum ALP	134.4 (78.27)	137.8 (84.6)	123.2 (53.1)	0.103
Serum CRP	2.57 (5.85)	1.6 (2.5)	5.7 (10.7)	<0.001
Hospital LOS of 1 day	39 (57.4)	18 (34.6)	11 (68.8)	0.016

On the Chi-square test, there was no statistical difference between the two groups in terms of ultrasonographic difficulty (p>0.05). However, we found a Kappa value of -0.127 (p=0.275) corresponding to a strong disagreement between the intraoperative and ultrasonographic difficulty (Table 2).

**Table 2:** Comparison of Ultrasonographic Difficulty Between The Easy LC And Difficult LC Groups LC= Laparoscopic Cholecystectomy

	Intraoperative Easy LC, n = 52	Intraoperative Difficult LC, n = 16	p-value
Ultrasonographic Easy LC	43 (82.7)	15 (93.8)	0.275
Ultrasonographic Difficult LC	9 (17.3)	1 (6.3)	

## Discussion

Despite technological advancement, ultrasonography remains the first-line diagnostic test for cholelithiasis due to it non-invasive nature, widespread availability, economic feasibility, and high sensitivity for diagnosing cholelithiasis [17]. Ultrasonography enables the assessment of various crucial parameters, including gallbladder wall thickness, gallstone presence, size, common bile duct diameter, and liver dimensions. Taking advantage of these characteristics, studies have shown the use of ultrasound in predicting the intraoperative difficulty of LC [10]. However, in our study, we saw a significant disagreement between radiologic difficulty and intra-operative difficulty of LC.

LC involves intra-abdominal access, identification of the gallbladder, identification and ligation of the cystic duct and vessels, and finally dissection of the gallbladder from the gallbladder fossa [7]. In most cases, it is a straightforward procedure. However, in some cases, this surgery may be prolonged and or complicated due to obstructed view, dense adhesions, or damage to the surrounding structures [10]. This is termed a difficult LC.

The current study saw a significantly high rate of LC among women with a mean age of 42 years, and no difference in terms of gender or age distribution based on intra-operative cholecystectomy. These findings were consistent with previous studies [5-10]. Furthermore, there was no difference in the rates of comorbid conditions including hypertension, diabetes mellitus, ischemic heart disease, and obesity, or presenting signs and symptoms. Yet, it is noteworthy that nearly a quarter of the patients in the present study suffered from acute pancreatitis. Though this has been described as a predictor of difficult LC, we did not see any difference among the two groups in terms of acute pancreatitis occurrence [18]. This might be attributed to various factors. Pancreatitis results in intra-abdominal inflammation and adhesions. As with any inflammatory response, chronic and recurrent inflammation might result in dense adhesions that result in difficult intra-abdominal surgeries. Although we did not report the number, onset, and duration of pancreatitis, all our patients suffered from an acute episode. Further studies are needed to investigate the role of pancreatitis in difficult LC and the interventions to overcome this.

Multiple predictors of difficult LC have been reported and studies have also shown some ultrasonographic criteria to predict the difficulty and have given scoring systems based on these findings [10]. These variables include gallbladder wall thickness, presence of impacted stones, common bile duct diameter, peri-cholecystic collection, stone count, and liver size. Similar findings have been replicated in larger-scale studies [10,19,20], reinforcing the potential for pre-operative predictions based solely on these six parameters extracted from abdominal ultrasound scans. However, these effects were not seen in the current study. This might indicate the inadequacy of this scoring system in predicting difficult LC in a varied clinical environment. Importantly, pure sonographic criteria might not be as useful as was reported in the original study as well. Further studies should focus on introducing a scoring system based on a combination of clinical, laboratory, and ultrasonographic predictors.

Another potential predictor of difficult LC is the peri-operative laboratory findings. Although there was no difference among our study groups in terms of the TLC, serum ALT or ALP, the difficult LC group had significantly higher mean CRP and mean bilirubin levels. These findings indicate an acute inflammatory response and bile duct obstruction which correlate with intra-operative difficulty. These findings align with previous investigations that have established similar associations [14,21,18]. Finally, the present study highlights that 57% of patients were discharged on the first post-operative day, with no difference based on operative difficulty. This might correlate with the importance of peri- and post-operative care of these patients which can affect the outcomes of patients undergoing cholecystectomy.

Limitations

We would like to acknowledge that the study limitations included the inability to employ the same doctor to perform all ultrasounds, a small sample size, and a lack of resources in the department.

## Conclusions

Despite its role in diagnosing cholelithiasis, the current study did not find any role of ultrasonographic assessment in predicting difficult LC. We believe a standardized scoring system can prove immensely useful as ultrasound is an easily available assessment tool in urban as well as rural areas. Further studies are thus required to develop such a scoring system for predicting difficult LC based on clinical, laboratory, and ultrasonographic assessment.
